# Successfully salvaging a HIV-positive patient with mixed CIDP and meningoencephalitis: a case report

**DOI:** 10.3389/fmed.2025.1537160

**Published:** 2025-03-11

**Authors:** Wen Wang, Jun Yang, Xinchao Liu, Ying Wen

**Affiliations:** ^1^Department of Infectious Diseases, The First Affiliated Hospital of China Medical University, Shenyang, China; ^2^Neurology Department, The First Affiliated Hospital of China Medical University, Shenyang, China; ^3^Infectious Disease Department, Peking Union Medical College Hospital, Beijing, China

**Keywords:** cytomegalovirus, meningoencephalitis, chronic inflammatory demyelinating polyradiculoneuropathy, ganciclovir resistance, acquired immunodeficiency syndrome

## Abstract

We describe an acquired immunodeficiency syndrome (AIDS) patient who first exhibited chronic inflammatory demyelinating polyneuropathy (CIDP) and subsequently developed meningoencephalitis due to ganciclovir (GCV)-resistant cytomegalovirus (CMV) infection. The patient first presented with peripheral nervous system (PNS) symptoms, followed by central nervous system (CNS) symptoms. Based on auxiliary examinations, including cerebrospinal fluid (CSF) tests, electromyography (EMG), brain magnetic resonance imaging (MRI) and GCV drug resistance tests, the patient was diagnosed with CIDP and meningoencephalitis due to CSF GCV-resistant CMV. After the combined application of intravenous immunoglobulin treatment, corticosteroid treatment, antiretroviral therapy (ART), and adjusted anti-CMV treatment, the patient achieved persistent relief. This case underscores the importance of considering CMV as a common etiology of neurological disorders in AIDS patients. It also highlights the necessity of prompt drug resistance testing when anti-CMV therapy yields suboptimal responses.

## Introduction

Cytomegalovirus (CMV) is a common pathogenic agent in central nervous system (CNS) opportunistic infections, and CMV meningoencephalitis is a major cause of mortality in acquired immunodeficiency syndrome (AIDS) patients (1, 2). CMV is also associated with peripheral nervous system (PNS) dysfunction in AIDS patients (3). As the first-line antiviral agent, ganciclovir (GCV) resistance primarily arises from mutations in the UL97 kinase gene (phosphotransferase) and/or UL54 DNA polymerase gene. Although primary resistance (pre-therapy mutations) is rare, secondary resistance due to prolonged antiviral exposure is increasingly reported in immunocompromised hosts (4). While drug-resistant CMV infections in the HIV-positive population are uncommon, the prevalence of GCV resistance among the HIV-positive population is the highest, followed by that of the organ transplant population (5). Resistance testing, including genotypic assays for UL97 and UL54 mutations, is critical for guiding therapy in refractory cases. Here, we report an AIDS patient with a concurrent neurological complications caused by GCV-resistant CMV.

## Case description

A 49-year-old male presented with bilateral lower limb weakness on March 1, 2022 that had existed for the prior 2 months. The patient was conscious and had no obvious sensory disorders, however, the muscle strength of both lower limbs decreased. Muscle atrophy of both lower limbs was noted, and the reflex of both knee tendons was weakened. The results of laboratory examinations were as follows: HIV-Ab (+), serum HIV RNA concentration of 1.92 × 10^6^ copies/mL, and CD4^+^ T-cell count of 6/μL. Lumbar puncture was performed, and cerebrospinal fluid (CSF) analysis revealed the following results: WBC count, 276 × 10^9^/L (0–8); protein concentration, 1.4 g/L (0.12–0.6); normal glucose and chloride concentration. The concentration of CSF CMV-DNA was 3 × 10^6^ copies/mL, while the CSF HIV-1 viral load was 2.89 × 10^5^ copies/mL (Table 1). Initial brain magnetic resonance imaging (MRI) revealed no significant lesions. An electromyogram (EMG) showed neurogenic damage. The motor nerve conduction velocities of the left and right median nerves, the right common peroneal nerve, and the left and right tibial nerves decreased as the distal latency increased. The patient met the electrodiagnostic criteria for chronic inflammatory demyelinating polyradiculoneuropathy (CIDP) recommended by the European Academy of Neurology/Peripheral Nerve Society (6). The patient was diagnosed with AIDS, CMV-related peripheral neuropathy and meningitis and was treated with GCV, intravenous immunoglobulin, methylprednisolone. Antiretroviral therapy (ART) (bictegravir sodium, emtricitabine, and tenofovir alafenamide fumarate tablets) was initiated 15 days after anti-CMV therapy. After treatment, the muscle strength of the patient gradually improved, and his CSF cell count decreased, but his CSF CMV DNA test remained positive after 2 months of anti-CMV treatment (Table 1). The coadministration of GCV and foscarnet was used as an adjusted anti-CMV regimen, while methylprednisolone was then tapered.

**Table 1:** Laboratory examination and treatment of the patient.

Date of examination and treatment	Reference range	Onset	2 months	3 months	8 months	1 year
Event		Decreased lower limb muscle strength	Muscle strength improved	Consciousness disorders, muscle weakness	UL97 gene mutation	Mental and muscular recovery, CSF HIV escape
CSF cell count (×10^6^/L)	0–8	276	10	50	16	31
CSF protein (mg/L)	120–600	1,415	1,142	1,990	1,010	1,441
Lumbar puncture pressure(mmH_2_O)	80–180	135	180	100	120	236
CSF HIV-RNA (copies/mL)	<20	2.89 × 10^5^	253	1,651	<20	1,210
CSF CMV-DNA (copies/mL)	<1.0 × 10^3^	3.04 × 10^6^	8.36 × 10^4^	2.30 × 10^3^	3.30 × 10^4^	<1.0 × 10^3^
CSF mNGS	0	CMV 2402series				CMV 279series
Plasma CD4+ T cell count (cells/uL)	410–1,590	6	18	22	11	148
Plasma CD8+ T cell count(cells/uL)	190–1,140	471	356	419	317	1,703
Plasma HIV-RNA (copies/mL)	<20	1.92 × 10^6^	90.1	75	<20	141
Serum CMV-DNA (copies/mL)	<1.0 × 10^3^	1.22 × 10^5^	4.26 × 10^3^	8.60 × 10^4^	<1.0 × 10^3^	<1.0 × 10^3^
ART regimen		B/F/TAF	B/F/TAF	F/TAF + DTG	F/TAF + DTG	F/TAF + DTG
Corticosteroid therapy		Methylprednisolone, 40 mg twice per day	Methylprednisolone, 12 mg once per day	Methylprednisolone, 20 mg twice per day	Hydrocortisone, 30 mg each morning and 20 mg each afternoon	Hydrocortisone, 15 mg each morning and 5 mg each afternoon
Anti-CMV treatment		Ganciclovir	Ganciclovir and foscarnet	Ganciclovir and foscarnet	Foscarnet	Foscarnet

CSF, cerebrospinal fluid; CMV, Cytomegalovirus; mNGS, metagenomics next-generation sequencing; ART, antiretroviral therapy; B/F/TAF, bictegravir sodium, emtricitabine and tenofovir alafenamide fumarate tablets; F/TAF, emtricitabine and tenofovir alafenamide fumarate tablets; DTG, dolutegravir.

**Figure 1:**
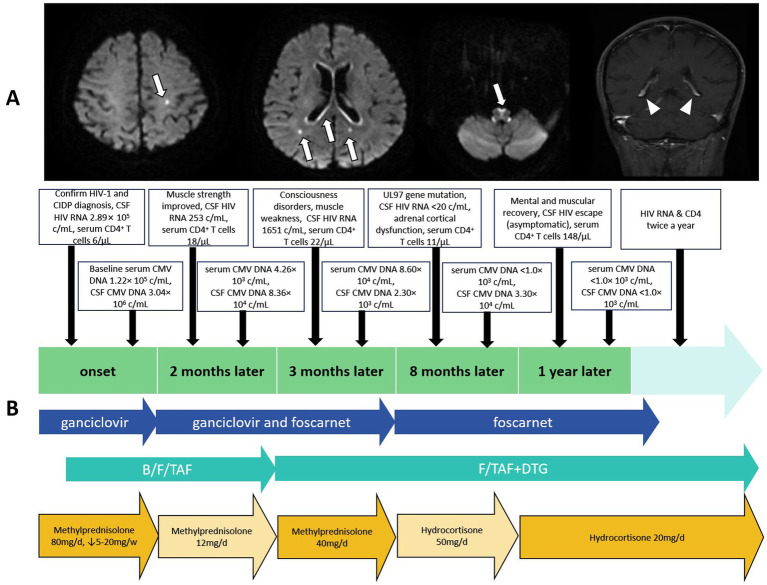
**(A)** Brain magnetic resonance imaging (MRI) of the patient. Diffusion-weighted imaging sequence revealed high signals in the left half of the oval center, near the posterior horn of the bilateral ventricles, bilateral ventricular walls, and anterior edge of the medulla oblongata (white arrows). The coronal image showed abnormal enhancement of the bilateral ventricles (Triangles). **(B)** The timeline for treatment adjustment and follow-up of this patient.

Three months later, the patient suffered from headache, ataxia, muscle weakness, and temporal disorientation. The Mini-Mental State Examination (MMSE) score was 18, and the CMV DNA and HIV RNA in CSF remained positive (Table 1). No mutation in the UL97 gene of CMV in the serum was detected by Sequence-Specific PCR followed by Sanger sequencing. Regrettably, due to the limitations of detection technology, we could not detect UL54 gene mutations in blood samples, nor could we detect UL97 and UL54 gene mutations in CSF. Diffusion weighted imaging (DWI) of the brain MR images were consistent with the diagnosis of CMV meningoencephalitis (Figure 1A). The coadministration of GCV and foscarnet continued, while bictegravir sodium was replaced by dolutegravir to increase the penetration of ART into the CNS. The patient became negative for CSF HIV RNA 2 weeks later. The patient’s neurological symptoms gradually improved in 2 months.

Eight months later, the patient’s MMSE score increased from 18 to 27, and his muscle strength also recovered. However, his CSF remained positive for CMV DNA. The A594V mutation in the UL97 gene of CMV in the CSF was detected by Sequence-Specific PCR followed by Sanger sequencing, but no drug resistance mutations were detected in the UL54 gene. GCV treatment was stopped, and foscarnet treatment was continued (Table 1). Moreover, hormonal testing revealed adrenal cortical dysfunction (data not shown).

After 1 year of treatment, the patient exhibited normal consciousness and muscle strength, CMV DNA was undetectable in CSF by PCR (<1,000 copies/mL), his CD4^+^ T-cell count was 148/μL (Table 1), and the patient’s brain MRI and electromyography also showed significant improvement (Figure 1A). The patient subsequently received reduced corticosteroids for CIDP. Due to adrenal cortex dysfunction, the patient received 20 mg/d hydrocortisone for maintenance treatment. Anti-CMV treatment was discontinued after 15 months of onset. At the three-year follow-up, the patient reached remission, with normal consciousness and muscle strength. The timeline for treatment adjustment and follow-up of this patient is clearly outlined in Figure 1B.

## Discussion

This patient had simultaneous involvement of the PNS and CNS, which is associated with CSF GCV-resistant CMV infection. The patient had no prior GCV exposure, and CMV resistance occurred during anti-CMV therapy, which suggested secondary CMV resistance rather than a primary drug-resistant CMV infection. Initial response to GCV was favorable, with reduced CMV DNA in serum and CSF, decreased CSF cell count, and improved peripheral neuropathy. However, by the third month, CMV DNA persisted in serum and CSF, and serum levels increased. UL97 resistance testing in blood was negative, prompting continued GCV and foscarnet therapy. By the eighth month, serum CMV DNA was undetectable, but CSF levels remained elevated, and the A594V mutation in the UL97 gene was detected in CSF CMV. This suggests resistance developed due to prolonged CMV infection and GCV therapy. Despite rapid HIV suppression with ART, inadequate immune restoration also contributed to poor anti-CMV response.

HIV and CMV infections, especially CMV infections, might play a key role in CIDP (7, 8). CIDP most likely results from an immunopathogenic mechanism, reflecting altered immune regulation caused by HIV and CMV infections. CIDP is triggered by an immune stimulus such as an infection (9). The relationship between CIDP and CMV infection in this case is complex. While CIDP is typically considered an immune-mediated disorder, the presence of CMV in the CSF suggests that CMV may have directly contributed to peripheral nerve dysfunction. This dual pathology complicates the decision to initiate corticosteroids, as their use in the setting of active CMV infection carries risks of exacerbating viral replication. However, in this case, the clinical improvement following corticosteroid administration supports the hypothesis that CIDP was primarily immune-mediated, with CMV acting as a cofactor. CSF pleocytosis could be a feature of HIV-positive CIDP patients (10, 11). For our patient, concomitant CMV infection might be another reason for CSF pleocytosis.

In AIDS patients with cryptococcal meningitis, ART initiation generally is deferred for 4 to 6 weeks after antifungal agents are started to avoid immune reconstitution inflammatory syndrome (IRIS). However, for CMV-related conditions, ART is usually started within 1–2 weeks of anti-CMV therapy. In this patient, ART was initiated 15 days after anti-CMV treatment, and no IRIS occurred. The subsequent encephalitis was consistent with CMV ventriculoencephalitis rather than IRIS, and corticosteroid use likely reduced IRIS risk.

For CIDP treatment, intravenous immunoglobulin, plasma exchange, and corticosteroid were recommended. AIDS patients with CIDP are generally younger, more steroid-responsive, and have a monophasic progressive course compared to HIV-negative patients (12). In treating CMV infection in AIDS patients, although the optimal duration of initial therapies has not been established, the combination of GCV and foscarnet is the preferred regimen. Except for GCV and foscarnet, other anti-CMV drugs were unavailable or difficult to obtain in China. The intravenous infusion of CMV-cytotoxic T lymphocytes (CTLs) and anti-CMV specific immunoglobulins were only reported in transplant patients (13). Continuous CMV disease progression after multiple weeks of GCV therapy can be an indication of drug resistance. Although the correlation between CMV genetic resistance and phenotypic resistance is undetermined and optimal testing methods are nonuniform, the detection of CMV genetic resistance still facilitates the improvement of anti-CMV therapies (14).

## Conclusion

In conclusion, patients with refractory CMV-associated nervous system disease progression should be tested for CMV drug resistance using CSF samples. In addition to GCV and foscarnet, other new anti-CMV drugs such as Maribavir will require being validated in HIV-positive population. Optimized therapies should include adjusting anti-CMV drugs, prolonging the treatment period and improving the immune status. Long-term maintenance therapy is also needed.

## Data Availability

The original contributions presented in the study are included in the article/supplementary material, further inquiries can be directed to the corresponding author.
